# A dose-response study of aerobic training for oxygen uptake, oxidative stress and cardiac autonomic function in type 2 diabetes mellitus: study protocol for a randomized controlled trial

**DOI:** 10.1186/s13063-018-2671-y

**Published:** 2018-05-24

**Authors:** Shalini Verma, Jamal Ali Moiz, Shahnawaz Anwer, Ahmad H. Alghadir, Mohammed Ejaz Hussain

**Affiliations:** 1 Centre for Physiotherapy and Rehabilitation Sciences, Jamia Millia Islamia (Central University), New Delhi, 110025 India; 2 Department of Rehabilitation, College of Applied Medical Sciences, King Saud University, Riyadh, Saudi Arabia

**Keywords:** Heart rate variability, Metabolic control, Cardiopulmonary fitness, Aerobic training, Oxygen uptake, Diabetes

## Abstract

**Background:**

Cardiac autonomic neuropathy is a commonly overlooked complication of type 2 diabetes mellitus (T2DM) characterized by an imbalance between sympathetic and parasympathetic supply to the heart, which contributes to cardiovascular morbidity and mortality. T2DM has also been shown to negatively influence oxygen kinetics and increase oxidative stress, which may be linked to the development of various chronic complications. Aerobic training has been reported to improve oxygen uptake, antioxidant defense, and cardiac autonomic function in T2DM; however, the effects of varying doses of exercise on these variables are not known. Therefore, the aim of the present study is to explore the effects of manipulating training variables (volume and intensity) on the regulation of oxygen uptake response, oxidative stress, and cardiac autonomic function in patients with T2DM.

**Methods:**

We will recruit 60 patients with T2DM, who will be randomly allocated into one of the three aerobic training groups: low-intensity, low-volume training; low-intensity, high volume-training; high-intensity, high-volume training; or to the control group receiving no supervised exercise. All participants will be assessed for the rate of oxygen uptake, levels of antioxidant enzymes and cardiac autonomic function at baseline and after 12 weeks of training. Secondary outcome measures will include cardiometabolic risk factors and body composition.

**Discussion:**

Despite a large body of evidence on the efficacy of aerobic training in the prevention and treatment of T2DM, there is no unequivocal exercise prescription for the same. Oxygen kinetics and oxidative stress are highly sensitive to the magnitude of physical activity. It would therefore, be interesting to study their interaction with chronic exposure to various doses of exercises and explore the optimal volume and intensity to bring about improvements in these parameters.

**Trial registration:**

Clinical Trials Registry – India, CTRI2017/08/009459. Registered on 23 August 2017. Retrospectively registered.

**Electronic supplementary material:**

The online version of this article (10.1186/s13063-018-2671-y) contains supplementary material, which is available to authorized users.

## Background

The current worldwide prevalence of diabetes mellitus is 425 million and is projected to reach 628 million by the year 2045 [1], with type 2 diabetes mellitus (T2DM) accounting for at least 90% of the cases [2]. T2DM is associated with dysfunction of various organs especially the heart and peripheral blood vessels, making it a multi-faceted metabolic disorder, with twofold to fourfold higher incidence of cardiovascular disease compared to patients without diabetes mellitus [3]. Diabetes mellitus has been reported to affect oxygen consumption causing a blunting of both peak and dynamic responses of oxygen uptake implying impairment of the control of oxygen delivery to and/or utilization of oxygen by contracting muscles [4]. In addition, hyperglycemia-induced oxidative stress is one of the major focuses of recent research related to diabetes mellitus [5]. Growing evidence indicates that oxidative stress is increased in diabetes mellitus because of the overproduction of reactive oxygen species (ROS) and the decreased efficiency of antioxidant defenses [6–8] and may be linked to the development of chronic complications of diabetes mellitus [9, 10]. Mechanisms that contribute to the formation of free radicals in diabetes mellitus may include not only increased non-enzymatic and auto-oxidative glycosylation, but also metabolic stress resulting from changes in energy metabolism, the levels of inflammatory mediators, and the status of antioxidant defense systems [11, 12]. Another commonly overlooked complication of T2DM, cardiac autonomic neuropathy (CAN) is characterized by an imbalance between the sympathetic and parasympathetic supply to the heart, which contributes to cardiovascular morbidity and mortality [13]. The pathogenesis of diabetic CAN is complex and involves a series of pathways activated by hyperglycemia resulting in increased oxidative stress, which can cause direct neuronal dysfunction or, endothelial dysfunction resulting in neuronal ischemia and cellular death [14].

### Aerobic training in diabetes mellitus

Considering the predominance of an inactive lifestyle in modern-day societies, exercise training constitutes the cornerstone of cardiovascular disease prevention in the diabetic population. Aerobic exercise is known to reduce weight and maintain good glycemic control, and thus reduce the risk of cardiovascular disease among diabetic patients [15]. The beneficial effects of aerobic training on the metabolic profile include significant reductions in indices of obesity (reduction in body mass index (BMI), total fat mass, sum of skin fold measurements, waist circumference and/or waist-to-hip ratio), improved cardiovascular risk profiles (increase in high-density lipoprotein/low-density lipoprotein cholesterol (HDL-C/LDL-C) ratio, normalization of lipid profiles, lowered blood pressure (BP) and resting heart rate, improved cardiac output and oxygen extraction), increase in the (sub)maximal aerobic exercise capacity, a significant decrease in glycemia and significant increase in insulin sensitivity, insulin secretion, and/or reduced glycosylated hemoglobin (HbA1c) concentrations [16–20]. It also improves skeletal muscle capitalization and blood flow, muscular glucose transporter 4 (GLUT4) levels, hexokinase, and glycogen synthase activities [21].

**Fig. 1:**
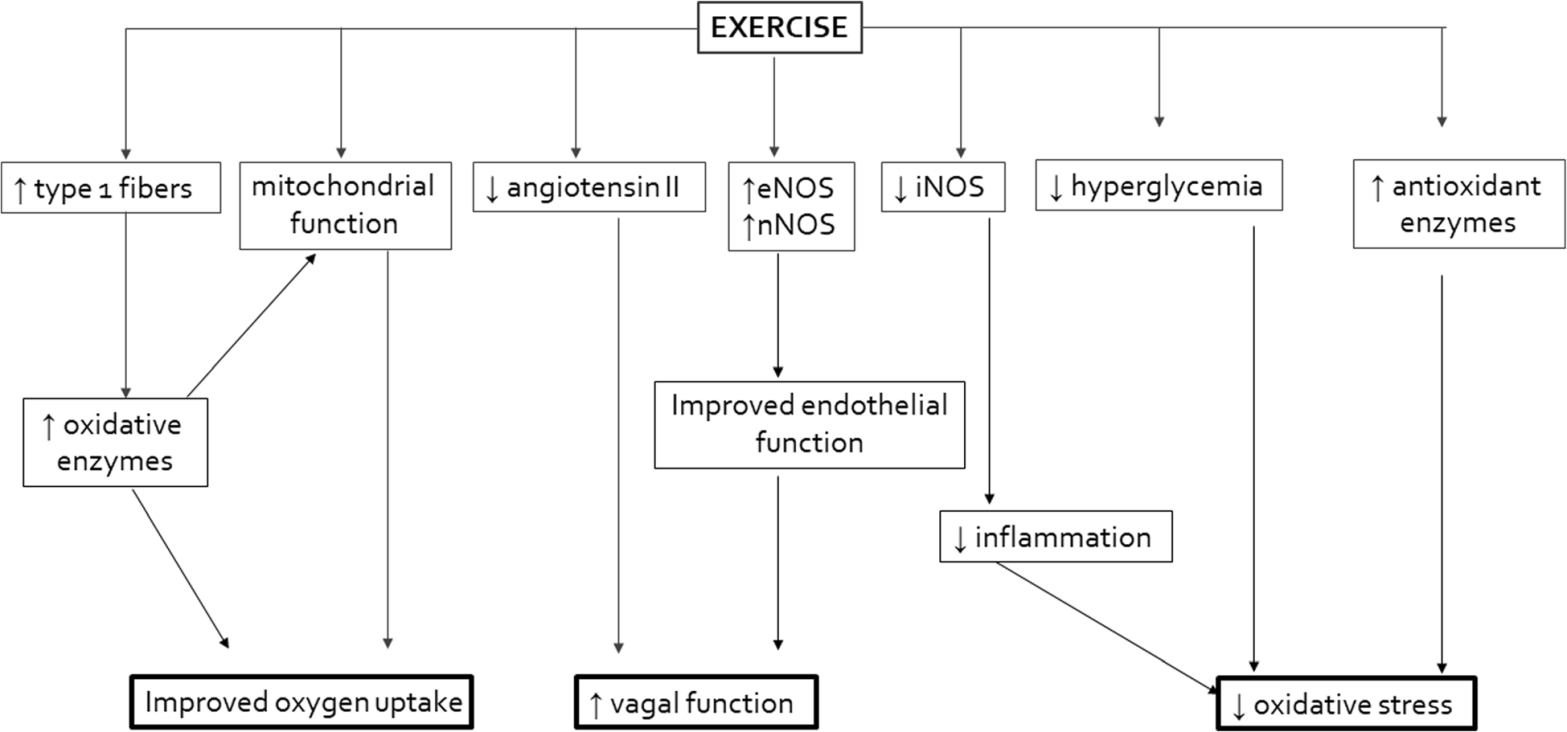
Exercise-mediated impact on oxygen uptake, oxidative stress, and cardiac autonomic function. ↓ downregulation/suppression, ↑ upregulation/stimulation, *eNOS* endothelial nitric oxide synthase, *nNOS* neuronal nitric oxide synthase, *iNOS* inducible nitric oxide synthase

In addition, aerobic training has been shown to improve oxygen uptake kinetic responses [22]. It has also been proven to enhance resting vagal activity and depress the sympathetic outflow [23] and recent reviews have emphasized its role in significant improvement in cardiac autonomic function in T2DM [24, 25]. Furthermore, Oliveira et al. [26] showed that an aerobic training program in subjects with T2DM resulted in important upregulation of antioxidant enzyme levels and increased nitric oxide bioavailability, which may help minimize oxidative stress and the development of the chronic complications of diabetes mellitus. The exercise-induced oxidative stress may function in a manner similar to the general principles of exercise training. That is, in order for an adaptation to occur, the physiological stimulus applied (in this case, ROS and reactive nitrogen species (RNS) production) must exceed a certain minimal threshold, effectively overloading the system. If overload is achieved, the physiological capacity of the body will expand or adapt, ultimately leading to improvement in health and/or human performance [27]. It remains to be elucidated however, what dose (volume and intensity) of aerobic exercise can compensate or even super-compensate for ROS production in patients with T2DM. Figure 1 outlines the proposed mechanisms through which aerobic training might improve oxygen uptake, oxidative stress, and autonomic modulation.

### Research objective and hypothesis

Although the American Diabetes Association (ADA) recommends at least 150 min/week of moderate-intensity aerobic physical activity or at least 90 min/week of vigorous aerobic exercise distributed over at least 3 days/week and with no more than 2 days consecutively without physical activity [28], a dose- response relationship with health benefits remains unclear. Therefore, the present study aims to evaluate the dosimetry of aerobic training programs in the regulation of oxygen kinetics, oxidative stress, cardiac autonomic function, and cardiometabolic parameters in T2DM. We hypothesize that varying the volume and intensity of aerobic training will result in a significant difference in oxygen uptake, oxidative stress, and cardiac autonomic function in patients with diabetes mellitus.

## Methods

### Study design

**Fig. 2:**
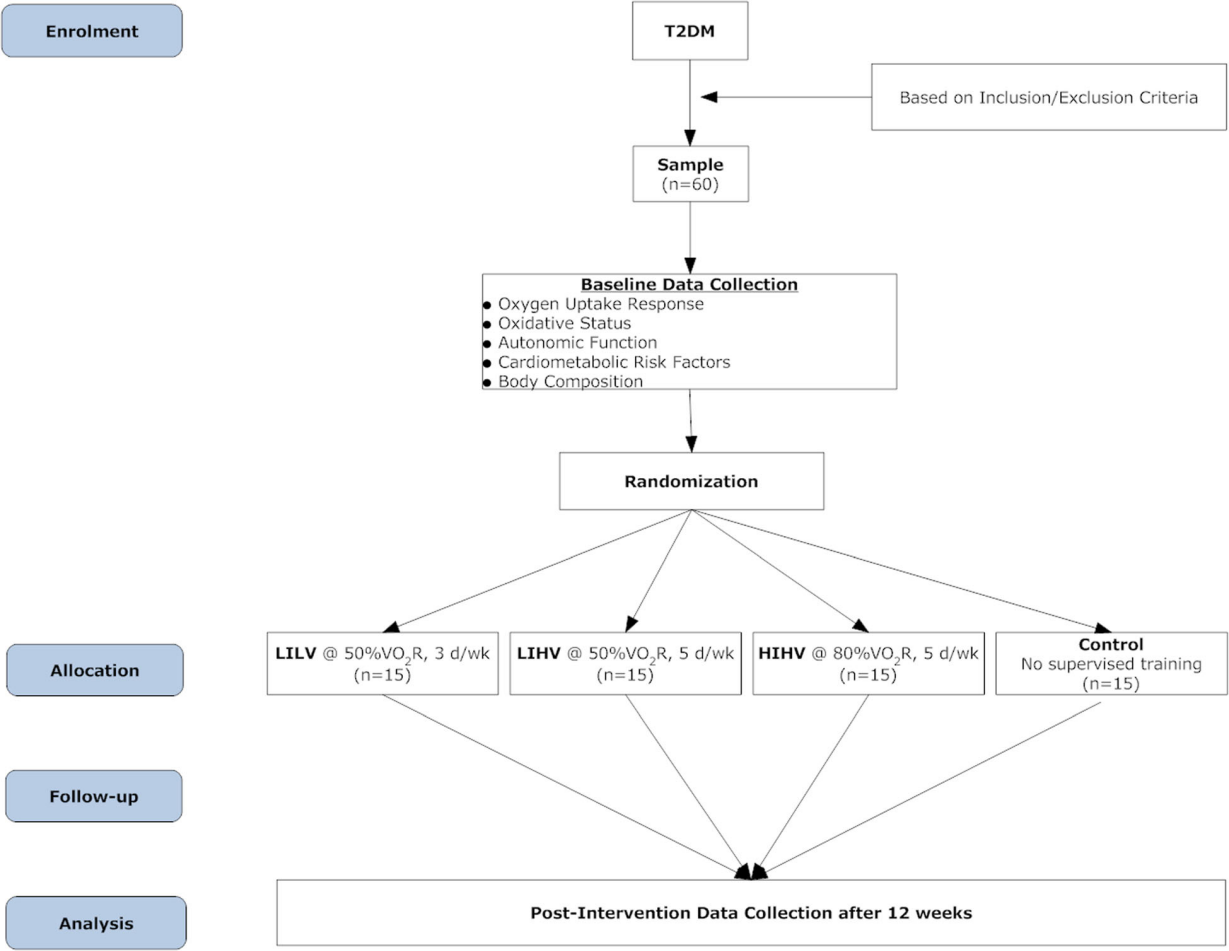
Flowchart of study design

**Fig. 3:**
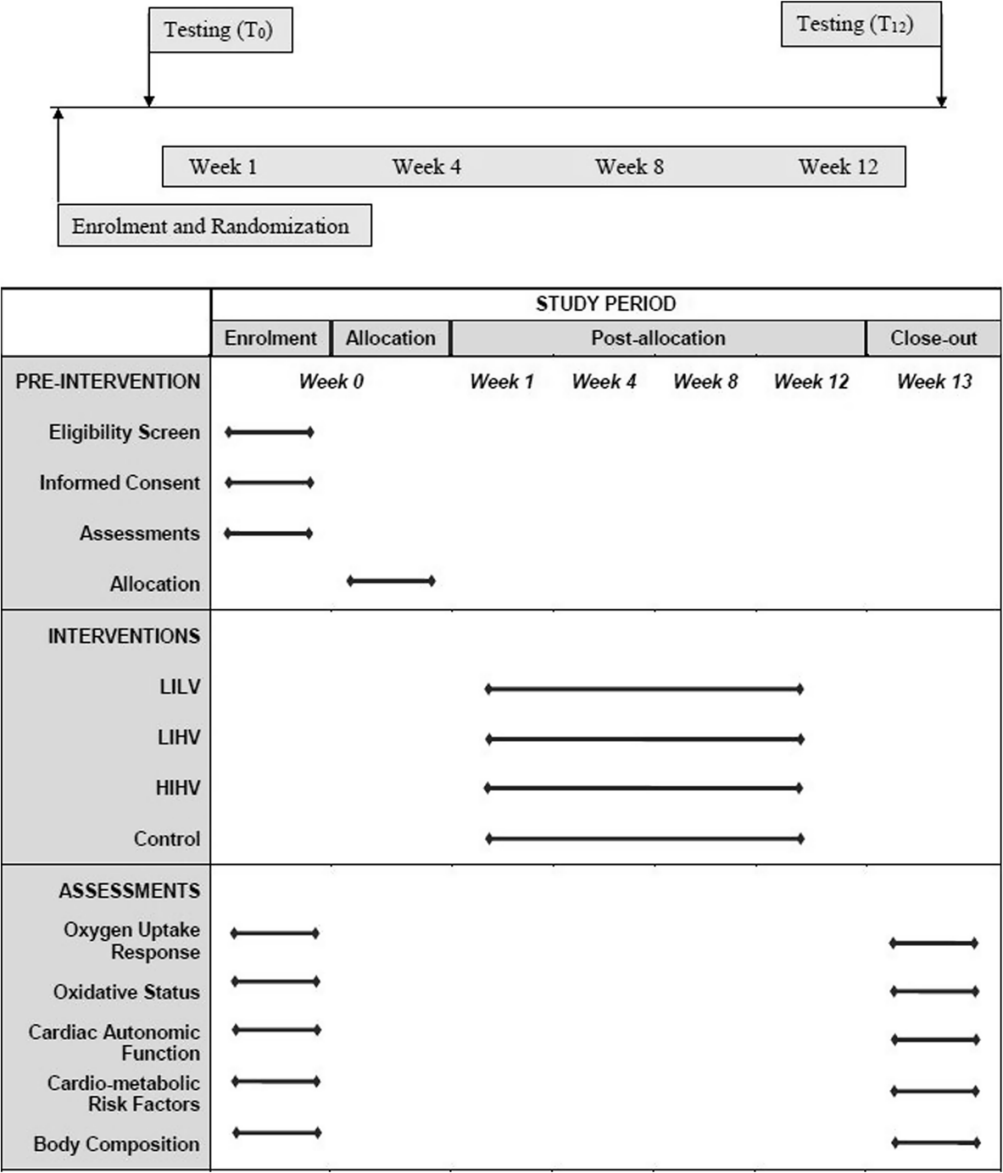
Standard Protocol Items: Recommendations for Interventional Trials (SPIRIT): Specific research plan and implementation steps. *LILV* low-intensity, low-volume training; *LIHV* low-intensity, high-volume training; *HIHV* high-intensity, high-volume training

The study is a single-blinded randomized controlled trial (with blinding of the outcome assessor) evaluating the dose response of aerobic training in patients with T2DM. It is a four-arm comparative pretest-posttest experimental design with random allocation of subjects into groups using a lottery method (Fig. 2). This trial has been designed in accordance with the Consolidated Standards of Reporting Trials (CONSORT) statement and is reported as per the Standard Protocol Items: Recommendations for Interventional Trials (SPIRIT) statement (Fig. 3). Additional file 1 shows this in more detail.

The study is approved by the Institutional Ethical Committee, Jamia Millia Islamia, New Delhi, India. All participants will be given an information sheet explaining the study purpose, methodology and their rights as research subjects. Written consent will be obtained.

### Participants

We will recruit 60 patients with T2DM from the university health center and nearby hospitals. Consenting participants will be randomly allocated into one of the three aerobic training groups: low-intensity, low-volume training (LILV); low-intensity, high volume-training (LIHV); high-intensity, high-volume training (HIHV), or to the control group receiving counselling and dietary advice but no supervised exercise. The inclusion and exclusion criteria are listed in Table 1.

**Table 1:** Inclusion and exclusion criteria for patient recruitment

Inclusion criteria	Age 30–70 years
	Diagnosis of type 2 diabetes mellitus ≥ 12 months
	Glycosylated hemoglobin (HbA1c) level 6.5–10%
	Receiving oral anti-diabetic medication
	Able to walk continuously for at least 20 min and climb one flight of stairs unaided without stopping
	Sedentary: do not engage in exercising more than 20 min on 3 or more days a week
Exclusion criteria	Body mass index > 40 kg/m^2^
	Exogenous insulin
	Autoimmune diseases
	Retinopathy, nephropathy, peripheral neuropathy
	Inflammatory and other conditions potentially associated with altered cytokine regulation
	Liver impairment
	Renal insufficiency (creatinine levels > 2.0 mg/dl)
	Diagnosed cardiovascular disease; recent onset of cardiac-origin symptoms
	Prescription of a very low caloric diet (fewer than 1000 kcal/day) or drugs for the treatment of obesity
	Orthopedic problems limiting physical activity
	Already engaging in intensive physical activity for > 2 h/week

### Recruitment method

The treating physician will screen (all) potential participants from the outpatient department of the Ansari Health Centre, Jamia Millia Islamia and inform them about the study. Potential participants interested in participating in the study will be referred to the Diabetes Rehabilitation Clinic where they will receive the participant information sheet and those meeting the inclusion criteria will be invited to participate in the trial. If they agree to participate, they will be required to sign a consent form and baseline data will be collected.

### Group allocation

Random allocation to one of the four groups will occur after confirmation of eligibility and baseline assessment. Blind allocation by a remote researcher will be performed using computer-generated random numbers. The concealment of allocation will be ensured by using sequentially numbered, sealed, and opaque envelopes.

### Procedures

Subjects with T2DM will be recruited based upon the inclusion and exclusion criteria and will be informed about the study. After explaining the purpose and methodology, participants will be given a consent form explaining their rights as research subjects. Following screening for eligibility, all participants will be required to visit the center on three separate days. Before each test, the subjects will be given a thorough demonstration. On the first day of testing, blood samples will be collected, and subjects will be assessed for body composition, resting blood pressure, and heart rate variability. On the second day of testing, the subjects will report to the Human Performance Laboratory, Centre for Physiotherapy and Rehabilitation Sciences (CPRS) where they will perform the graded maximal test and on day 3, they will perform the 20-min submaximal test (at 50% volume of oxygen consumed (VO_2_max)) to examine oxygen kinetics. After baseline testing, they will be allocated into one of the three aerobic training programs: low-intensity, low-volume training (LILV); low-intensity, high-volume training (LIHV); high-intensity, high-volume training (HIHV), or to the control group receiving counseling and dietary advice but no supervised exercise. All identifying information on the consent form and demographic/history questionnaire will be kept confidential by assigning a number to each subject.

### Interventions

Herein, VO_2_Reserve (VO_2_R) = VO_2_max – VO_2_rest, calculated during the graded maximal test for each subject. Participants will be randomly allocated into one of the four groups:

i. Low-intensity, low-volume training (LILV) 3 times/week performing treadmill walking at 50% VO_2_R
ii. Low-intensity, high-volume training (LIHV) 5 times/week performing treadmill walking at 50% VO_2_R
iii. High-intensity, high-volume training (HIHV) 5 times/week performing treadmill walking at 80% VO_2_R
iv. Control group receiving counseling and dietary advice but no supervised exercise.

### Outcomes

The primary and secondary outcomes are listed in Table 2. All outcomes will be assessed at baseline and after 12 weeks of intervention.

**Table 2:** Outcomes and criterion measures

Primary Outcomes	
Oxygen Uptake Response	
a) Maximal Response	Peak oxygen consumption
b) Dynamic Response	Time constant of rise to steady state (“on” kinetics) Time constant of recovery from steady state (“off” kinetics)
Oxidative stress	
a) Antioxidant enzymes	Catalase Superoxide dismutase Glutathione peroxidase
b) Reactive species	Nitric oxide
Cardiac Autonomic Function	
a) Resting	Heart Rate Variability Time-domain indices: AvgNN, SDNN, RMSSD, pNN50 Frequency-domain indices: Total power, LF, HF, LF: HF ratio
b) Post-exercise	Heart rate recovery at 1 (HRR_1min_) and 2 min (HRR_2min_)
Secondary Outcomes	
Cardiometabolic risk factors	
a) Glycemic control	Glycosylated hemoglobin Fasting blood glucose
b) Lipid profile	Total cholesterol High-density Lipoprotein Low-density Lipoprotein Triglycerides
c) Resting blood pressure	Systolic blood pressure Diastolic blood pressure
Body composition	
a) Body fat (%)	Percent body fat derived from sum of skinfolds
b) Central obesity	Waist circumference Waist-Hip ratio

*AvgNN* Mean of N-N intervals, *SDNN* Standard deviation of N-N intervals, *RMSSD* Square root of the mean squared differences between adjacent RR intervals, *pNN50* Percentage of interval differences of adjacent RR intervals greater than 50 milliseconds derived from differences between consecutive RR intervals, *LF* Low frequency power, *HF* High frequency power, *LF/HF ratio* Ratio of low and high frequency power

### Oxygen uptake

Oxygen uptake responses will be assessed using the maximal response (VO_2_ max) and dynamic response (oxygen kinetics).

- *Maximal response (VO*_*2*_*max)*: peak oxygen consumption will be measured using a graded exercise test using the Balke protocol [29] with concomitant recording of the electrocardiogram. During the pilot and feasibility analysis, this test was found to be better tolerated in the study population than the modified Bruce protocol. The treadmill test will be terminated if the participant
  i. Reaches his/her peak oxygen consumption or predicted maximum heart rate
  ii. Indicates that he/she cannot continue the testing
  iii. Has systolic blood pressure (SBP) above 220 mmHg or diastolic blood pressure (DBP) above 100 mmHg or
  iv. Develops abnormal electrocardiographic changes [30].
  The anaerobic threshold will be estimated during the maximal exercise test using the ventilatory threshold (VT). The VT is the workload at which the rate of pulmonary respiratory minute ventilation abruptly increases non-linearly during a progressive exercise test [31]. The VCO_2_ versus time and ventilatory equivalents (VE) versus time plots (AD instruments, Metabolic Module) will be used to graphically determine the VT.
- *Dynamic response (oxygen kinetics)*: oxygen kinetics describes the rate at which the cardiorespiratory system is able to deliver oxygen to the skeletal muscle and the rate at which oxygen is consumed by skeletal muscle. The rise to steady state is described by a time constant. A slowed time constant is a marker of impaired oxygen delivery/extraction. It takes longer for an individual with a slowed time constant to reach steady state [22].

Using a gas analyzer, VO_2_ will be recorded every 10 s during a submaximal exercise (20 min of walking at a treadmill speed predicted to elicit 50% VO_2_max). To assess the uptake kinetics, the time constant of the rise in VO_2_ from rest to steady state VO_2_ will be calculated.

A total of 10 min of controlled recovery will be calculated following the submaximal exercise to assess the off kinetics. The first part of the recovery will comprise a 1-min and 30-s standing phase, during which the subject is required to remain motionless and upright on the treadmill. Immediately following this, the subject will be seated for 8 min and 30 s, on a chair placed directly behind them on the treadmill. This combination of a short period of standing followed by seated recovery is similar to the protocol used in previous studies [32, 33]. The starting point of the curve will be forced to equal the average VO_2_ over the final 3 min of exercise to minimize the influence of spurious breathing at the end of the exercise on the span of the recovery curve. Excess post-exercise oxygen consumption (EPOC) will be calculated as the total area under the curve and the time constant will be estimated [33]. The time constants for both on and off kinetics will be calculated as a mono-exponential function.

### Oxidative stress

Blood samples will be collected from the antecubital vein after a 12-h fast and a 48-h period of no exercise. All samples will be taken in the morning to avoid the confounding effect of diurnal variation in oxidative stress parameters [34]. All samples will be analyzed in duplicate and then averaged. Catalase (CAT), superoxide dismutase (SOD) and glutathione peroxidase (GPX) activity in the plasma will be assessed using a commercial kit (Cayman Chemicals, Item no. 707002, 706,002, and 703,102, respectively) at wavelengths of 540 nm, 340 nm, and 450 nm, respectively. The nitric oxide (NO) levels will be measured as nitrites, using the Griess reaction and the absorbance will be read at 540 nm (Cayman Chemicals, Item no. 780001).

### Cardiac autonomic function

Cardiac autonomic function will be assessed as follows:

- *Heart rate recovery (HRR)*: heart rate recovery is a measure of the rate of decline of the heart rate during the first few minutes after peak exercise [35]. The heart rate will be derived from a continuous record obtained via electrocardiography (ECG) with surface electrodes placed in a lead-II arrangement. Lead site preparation and placement will be standardized according to American Heart Association standards. HRR will be calculated as the absolute difference between the heart rate at peak exercise and the heart rate recorded at 1- (HRR_1min_) and 2- min (HRR_2min_) post-exercise.
- *Heart rate variability (HRV)*: ECG signals will be recorded for 20 min with the participant in the supine position before the graded exercise test. Participants will be instructed to close their eyes and avoid any bodily movement or conversation during recording. Since the breathing rate has been found to confound HRV, breathing pace will be controlled at 12 breaths/minute with a metronome. After recording, all data will be stored and analyzed offline. Analysis of HRV will be performed using a time series of the last 5 minutes selected from the 20-min recording [36]. Data will be visually and automatically inspected for ectopic beats (premature, supraventricular, and ventricular); there must be no more than 10% ectopic beats in each record to be included in the analysis. Time and frequency domain variables of the HRV will be analyzed through the detection of R waves. ADInstruments LabChart version 7.3.7 software with HRV Module version 1.4.2 using Weltch window (Power Lab 8 SP, AD Instruments, Australia) will be used as the data acquisition software for recording ECG data, which calculates the R-R intervals as the measure of the difference between successive beats. All data acquisition and post-acquisition analyses will be performed in accordance with the guidelines proposed by the Task Force of the European Society of Cardiology and North American Society of Pacing and Electrophysiology [37].

### Cardiometabolic risk factors

Cardiometabolic risk factors will be assessed as follows:

- *Glycemic and lipid profiles*: HbA1c will be measured using 2–3 mL of blood drawn from participants who have fasted at least 10 h from the night before. HbA1c will be measured using high-performance liquid chromatography (HPLC). The fasting blood glucose and lipid profiles (comprising total cholesterol, triglycerides, HDL-C and LDL-C) will be estimated from serum samples. Total cholesterol and triglycerides will be measured using enzymatic colorimetric methods with cholesterol oxidase-peroxidase amino phenazone phenol and glycerol-3-phosphate oxidase-peroxidase amino phenazone phenol. HDL-C will be measured using a homogenous enzymatic colorimetric assay. LDL-C will be calculated using the Friedewald formula.
- *Resting blood pressure*: SBP and DBP will be measured using a standard sphygmomanometer. The participants will be strictly prohibited from consuming any caffeinated products 30 min before measurements; also exercising and smoking will be prohibited for the same duration before measurements. The patient will remain in relaxed seated state for 5 min and the BP cuff will be placed on either the right or left arm of the patient with the BP recording visible on the display. A second recording of the patient will be taken after 2 min. If the measurements display a BP difference ≥ 5 mmHg, further readings would be obtained until there are two consecutive stable measurements. An average of the two stable measurements will be considered the final reading. Following a rest period of 1–2 min, a recording from the other arm will be taken. If there is a measurement discrepancy between the upper extremities, then the extremity with the highest measurement will be used for analysis.

### Body composition

Body composition is defined as the amount and distribution of fat mass relative to the lean body mass. The amount (percentage) of body fat will be estimated using skinfold measurements and the distribution of fat (visceral adiposity/central obesity) will be assessed using waist circumference and waist-hip ratio.

Body density will be calculated from skinfold measurements taken at three sites (the chest, abdomen, and thigh in men; the triceps, thigh, and supra-iliac area in women), using the equations of Jackson and Pollack, and the Siri equation will be used to convert body density to percent body fat. Visceral obesity is defined as a waist circumference > 88 cm in women and > 102 cm in men. Waist circumference will be measured using a tape placed horizontally at the mid-point between the iliac crest and the lower aspect of the floating ribs in the mid axillary line, at the end of a normal expiration. Hip circumference will be measured at its greatest gluteal protuberance.

#### Sample size calculation

The number of subjects was determined using Software G. Power 3.192. An effect size of 0.44 was obtained from the data relative to changes in maximum aerobic capacity in a study by Lambers et al. [38], which examined the effects of aerobic training on obesity and cardiovascular risk in T2DM. A priori analysis of sample size using the *F* test (repeated measures analysis of variance (ANOVA), within-between interaction) showed that the total sample size required would be 52 to have 95% power at effect size of 0.44 and alpha level of 0.05. Considering the dropout rate from a previous similar investigation [30], the total sample size obtained is 60 or 15 subjects per group, using the allocation ratio of 1 for the four groups.

### Data monitoring

The researchers recruiting, implementing, and assessing the intervention will update the research team monthly about the study progress. No interim analysis will be performed.

### Harms

Aerobic training is an evidence-based intervention for glycemic control in patients with type 2 diabetes mellitus. All training sessions will be supervised by a professional physiotherapist to prevent any episode of hypoglycemia. However, if the patients experience a mild drop in blood sugar, it will be managed by trained professionals (oral administration of glucose as per American College of Sports Medicine guidelines). The doses of exercise proposed are in accordance with the guidelines of a joint position statement by the American Diabetic Association and American College of Sports Medicine. All potential adverse effects and unintended effects of the intervention will be reported.

### Auditing

The research supervisor (MEH) will randomly perform undisclosed laboratory visits during two assessments and one intervention session to assess protocol fidelity.

#### Statistical analyses

The intention-to-treat (ITT) analysis set will include all subjects who were randomized to the study and received at least one session of training. The normality of distribution will be examined for all variables and those found to have a non-normal distribution will be log transformed for further analyses. One-way ANOVA will be used to compare the groups at baseline. To test for the differences between groups and across two assessments, 4 × 2 split-plot ANOVA with group (LILV, LIHV, HIHV, control), time (baseline, 12 weeks) and interaction effect (Group × Time) will be employed. If the main effect of a group is found to be significant, the Bonferroni test will be employed as post hoc analysis. The significance level will be set at *p* < 0.05.

### Ethics

The trial methodology has been designed to incorporate features that minimize bias in controlled trials: randomization, concealed allocation, specification of eligibility criteria, blinded outcome assessment, blinded analysis, and ITT analysis.

This protocol was registered at the Clinical Trials Registry of India (CTRI/2017/08/009459) and was prospectively approved by the Institutional Ethical Committee, Jamia Millia Islamia (Reference:17/9/43/JMI/IEC/2015).

### Protocol amendments

Potential protocol modifications will be formally approved by the Institutional Ethical Committee before being implemented. The amendments will be communicated to the trial registries and outlined at the study dissemination.

## Discussion

Aerobic exercise training is an effective intervention for the prevention and treatment of insulin resistance and T2DM, but there is no unequivocal exercise prescription currently available. Investigations that have studied the effects of aerobic training in T2DM have used a spectrum of exercise volumes and intensities, but a comparison of these results in order to establish the most effective prescription remains unexplored. In addition, since the same amount of exercise training does not uniformly affect all cardiometabolic variables, a dose-response study would provide threshold dosimetry to bring about changes in the variable of interest and improve the specificity of the exercise prescription.

Oxygen kinetics and oxidative stress are highly sensitive to the magnitude of physical activity. The intensity of exercise is a significant determinant of (a) the profile of oxygen consumption influencing the presence/absence of a slow component and (b) the redox balance that impacts on the levels of oxidative stress. It would therefore be relevant to study their interaction with chronic exposure to various doses of exercise.

Another interesting facet of the study is exploring the response of NO, which might play a significant role in the regulation of oxygen uptake, oxidative stress, and cardiac autonomic function. The dual role of NO as a vasodilator and a competitive inhibitor of cytochrome-c inhibition of mitochondrial respiration, results in increased oxygen delivery but compromised utilization through the electron transport chain. This maintains high intramyocyte PO2 levels that are elevated during exercise and are speculated to affect oxygen kinetics. Superoxide reacts rapidly with NO, reducing NO bioactivity and producing the oxidative peroxynitrite radical. SOD catalyzes the conversion of superoxide to hydrogen peroxide, sparing NO. It also potentiates the exercise-induced eNOS expression through hydrogen peroxide. This suggests there is a close linear relationship between NO bioavailability and antioxidant enzymes. Paradoxically, due to its labile state, NO at high concentrations is also a source of oxidative stress. In addition, there is strong evidence for the presence of NOS distributed throughout cardiac autonomic neurons, but in studies its activity is equivocal. NO-mediated response to training is dependent on a host of factors including the levels of NOS expression and activity, severity of oxidative stress, NO binding to antioxidant molecules hemoglobin and glutathione [39], and individual patterns of physical activity [40, 41]. For these highly varied outcomes, evidence suggests the possible existence of an exercise amount/effort threshold pivotal for the regulation of NO production. However, the precise identification of these physical effort thresholds requires further study.

Exploring the optimal volume and intensity to bring about positive changes in oxygen consumption responses, oxidative status and cardiac autonomic function may hold important clinical implications and may help identify the underlying mechanisms of the differential effects of varying doses.

### Trial status

The trial is currently recruiting participants. Recruitment began in February 2016 and is anticipated to end in May 2018. The trial registration number is CTRI/2017/08/009459.

Registration date: 23 August 2017 registered retrospectively at Clinical Trials Registry - India (ctri.nic.in)

## Additional files

Additional file 1: SPIRIT 2013 Checklist: recommended items to address in a clinical trial protocol and related documents. (DOC 120 kb)

Additional file 2: Patient information sheet and consent form. (DOCX 20 kb)

## Abbreviations

ADA: American Diabetes Association
ANOVA: Analysis of variance
BMI: Body mass index
BP: Blood pressure
CONSORT: Consolidated Standards of reporting trials
ECG: Electrocardiography
EPOC: Excess post-exercise oxygen consumption
HbA1c: Glycosylated hemoglobin
HDL-C: High-density lipoprotein cholesterol
HIHV: High-intensity, high-volume training
HPLC: High-performance liquid chromatography
HRR: Heart rate recovery
HRV: Heart rate variability
ITT: Intention to treat
LDL-C: Low-density lipoprotein cholesterol
LIHV: Low-intensity, high volume training
LILV: Low-intensity, low-volume training
MHR: Maximal heart rate
NO: Nitric oxide
ROS: Reactive oxygen species
SOD: Superoxide dismutase
SPIRIT: Standard Protocol Items: Recommendations for Interventional Trials
T2DM: Type 2 diabetes mellitus
VO_2_: Volume of oxygen consumed
VO_2_max: Maximum volume of oxygen consumed
VO_2_R: Volume of oxygen consumed at reserve
VO_2_rest: Volume of oxygen consumed at rest
VT: Ventilatory threshold
