# Supplementary material for: A dose-response study of aerobic training for oxygen uptake, oxidative stress and cardiac autonomic function in type 2 diabetes mellitus: study protocol for a randomized controlled trial
Source: Trials. 2018 May 24;19:289. doi: 10.1186/s13063-018-2671-y (PMC5968520; doi:10.1186/s13063-018-2671-y)
Supplement: Supplementary file 2 — Patient information sheet and consent form. (DOCX 20 kb) [file 13063_2018_2671_MOESM2_ESM.docx]

**SUBJECT INFORMATION SHEET AND CONSENT FORM**

**Title of the project:** Aerobic Training and its effects on Oxygen Kinetics, Oxidative Stress and Cardiac Autonomic Function in Type 2 Diabetes Mellitus: A Dose-Response Study

**Site of the investigation:** Centre for Physiotherapy and Rehabilitation Sciences (C.P.R.S.),

Jamia Millia Islamia (J.M.I), New Delhi-110025.

**Name and address of the Principle Investigator:** Prof. Ejaz Hussain

Director, CPRS,

Jamia Millia Islamia

New Delhi- 110025

**Contact number of the Principle Investigator:** 9899339816

**Aim of the research:** To compare the effects of different doses of exercise on improvement in blood sugar in Type 2 Diabetes Mellitus.

**Methods of the research:** You will be distributed into one of the three 12-week exercise programs. Blood will be collected from you by a trained medical professional, prior to the beginning and after completion of 3 months training (treadmill running at different speeds) to test for blood glucose, lipid profile and antioxidant enzymes, at Ansari Health Center, Jamia Millia Islamia.

**Expected duration of the subject participation:** 3 months

**The benefits to be expected from the research to the subject or to others:** The exercise provided to you will result in improvement of blood sugar control, lipid profile and cardiovascular fitness.

**Alternative treatment/procedure options:** No alternative treatment/ procedure options will be provided to you.

**Right to prevent use of biological samples (DNA, cell line etc.) at any time during the research:** The study does not require the collection of any such biological sample from you.

**Any risk to the subject associated with the study:** All training sessions will be supervised by a professional physiotherapist to prevent any episode of low blood sugar. However, if you experience mild drop in blood sugar, it will be managed by trained professionals (as per, American College of Sports Medicine guidelines).

**Maintenance of confidentiality of records:** All the information provided by you will be kept strictly confidential. It will not be shared with any person without your consent.

**Provision of free treatment for research related injury:** Free treatment for research related injury will be provided to you if required, as per JMI rules.

**Compensation of subjects for disability or death resulting from such injury:** The study does not pose risk for the occurrence of any disability or death resulting from research related injury. In case of any such occurrence, compensation shall be provided as per JMI rules.

**Right to withdraw:** Your participation in this study is voluntary. You have the right to withdraw from the study at any time, without any penalty.

**Amount of clinical sample in quantity:** 4-5 ml of blood will be collected from you by a trained medical professional.

**Source of funding for the Investigation:** The study will be funded by Centre for Physiotherapy and Rehabilitation Sciences, Jamia Millia Islamia, New Delhi.

**In case of drug trials:** No drug will be given to you.

**Foreseeable extent for information:** Research data will be used for future studies and publication without revealing your identity.

**Risk of discovery of biologically sensitive information:** The study does not pose any risk of discovery of biologically sensitive information.

**Permission granted by the participant for photograph to be published**: You have the right to grant permission for publishing your photograph. You can choose any option. Yes ( ), no ( ).

**Responsibility of investigator:** All the sessions will be supervised by the researchers to ensure that correct technique is used. The investigator will comply as per the Consent Performa and will not force you at any part of the study.

**Consent**

1. I agree voluntarily to take part in this study.
2. I have been explained the purpose and other details of the study. I have been given a full explanation of the procedures involved.
3. I have been given an opportunity to ask questions and all my questions have been answered to my satisfaction.
4. I am free to withdraw from the study at any time without any reason and without my medical care or legal right being affected.
5. I understand that the information in my medical records is essential to evaluate the results of the study. I agree to release this information on the understanding that will be treated confidentially.
6. I agree that I will not be referred to by name in any reports/documents/any other means concerning this study.
7. I have been explained the risks and benefits for the patients and society associated with the study.
8. I agree that if I am harmed as result of taking part in the study, treatment will be provided free of cost by the PI/Institution/University.
9. I agree that the biological samples collected during this study may be stored for future use.

I willingly agree to take part in the above study.

Allow Do not allow

Signature of the Participant Date:

Name:

Age:

Address:

Signature of the Investigator: Date:

Signature of the Witness: Date:
